# Treatment of Type 2 Diabetes Mellitus in Advanced Chronic Kidney Disease for the Primary Care Physician

**DOI:** 10.7759/cureus.64663

**Published:** 2024-07-16

**Authors:** Mary Mallappallil, Sandeep Sasidharan, Jacob Sabu, Sabu John

**Affiliations:** 1 Internal Medicine and Nephrology, New York City (NYC) Health + Hospitals/Kings County Hospital Center, Brooklyn, USA; 2 Internal Medicine and Nephrology, State University of New York (SUNY) Downstate University of Health Sciences, Brooklyn, USA; 3 Internal Medicine, State University of New York (SUNY) Downstate Health Sciences University, Brooklyn, USA; 4 Internal Medicine and Cardiology, New York City (NYC) Health + Hospitals/Kings County Hospital Center, Brooklyn, USA; 5 Internal Medicine and Cardiology, State University of New York (SUNY) Downstate University of Health Sciences, Brooklyn, USA

**Keywords:** sglt2-inhibitors, insulin regimen, hd ( hemodialysis ), peritoneal dialysis, kidney transplantation, end stage renal disease, type 2 diabetes mellitus, chronic kidney disease

## Abstract

Diabetes mellitus (DM) is a common cause of chronic kidney disease (CKD), leading to the need for renal replacement therapy (RRT). RRT includes hemodialysis (HD), peritoneal dialysis (PD), kidney transplantation (KT), and medical management. As CKD advances, the management of DM may change as medication clearance, effectiveness, and side effects can be altered due to decreasing renal clearance. Medications like metformin that were safe to use early in CKD may build up toxic levels of metabolites in advanced CKD. Other medications, like sodium-glucose co-transporter 2 inhibitors, which work by excreting glucose in the urine, may not be able to work effectively in advanced CKD due to fewer working nephrons. Insulin breakdown may take longer, and both formulation and dosing may need to be changed to avoid hypoglycemia.

While DM control contributes to CKD progression, effective DM control continues to be important even after patients have been placed on RRT. Patients on RRT are frequently taken care of by a team of providers, including the primary care physician, both in and outside the hospital. Non-nephrologists who are involved with the care of a patient treated with RRT need to be adept at managing DM in this population. This paper aims to outline the management of type 2 DM in advanced CKD.

## Introduction and background

Epidemiology of CKD and DM

According to the United States Renal Data System, DM continues to be the most common cause of chronic kidney disease (CKD). The prevalence of CKD in adults has remained stable overall at 14%, while diabetes mellitus (DM) has increased in those with and without CKD, reaching 9.5% and 35.6%, respectively, in the time period between 2017 and 2020. CKD prevalence has risen in the groups of individuals who are older than 65 years, women, non-Hispanic black individuals, and those with DM, with the latter increasing from 34% to 38% in 2017-2020 compared to 2013-2016. There is a significant contribution to the cost of DM and cardiovascular disease (CVD) care in those with advanced CKD [1,2].

Hospital-acquired conditions (HAC) under the inpatient prospective payment system (IPPS) include manifestations of poor glycemic control: hospital-acquired diabetic ketoacidosis, nonketotic hyperosmolar coma, hypoglycemic coma, secondary diabetes with ketoacidosis, and secondary diabetes with hyperosmolarity [3]. Hence, effectively managing diabetes becomes significant for internists treating CKD patients due to the increasing overlap between these conditions [4-6].

Important points about insulin metabolism in advanced CKD

DM is a disease condition that causes alterations in insulin secretion. Insulin is a peptide hormone containing 51 amino acids and has a molecular mass of 5,808 Daltons. It is a heterodimer with alpha and beta chains linked by disulfide bridges. Intrinsic insulin is released into the portal circulation and is metabolized by the liver (75%) and kidney (25%). The kidney has a crucial role in the clearance and breakdown of circulating insulin based on glomerular filtration, reabsorption, endocytosis by tubular cells, and diffusion via the peritubular capillaries [7]. The complexity of the insulin molecule is due to its disulfide bonds, which prevent simple hydrolysis at the luminal proximal tubular brush border. Insulin requires endocytosis in tubular cells and degradation in lysosomes. Impairment of renal clearance due to CKD results in a longer half-life of circulating insulin, a decrease in the need for insulin therapy in those with DM, and better glycemic control, contributing to a condition called “burnt-out DM.” It is seen in one-third of patients with T2DM and is characterized by low or low-normal hemoglobin A1C (HbA1C <6%) despite discontinuing DM medications. Burnt-out DM may be explained by reduced renal insulin clearance and gluconeogenesis, along with diminished appetite in advanced CKD [8]. 

## Review

Diagnosis, monitoring, and targets

Diagnosis and Monitoring

Diabetes may be diagnosed based on plasma glucose criteria, either the fasting plasma glucose (FPG) value >125 mg/dL (7.0 mmol/L) or the two-hour plasma glucose (2-h PG) value >200 mg/dL (11.1 mmol/L) during a 75-g oral glucose tolerance test (OGTT) or hemoglobin A1c (HbA1C) >6.5% (48 mmol/mol), or in patients with classic symptoms of hyperglycemia or hyperglycemic crisis, a random plasma glucose ≥200 mg/dL (11.1 mmol/L) [9]. Alternate glycemic markers like glycated albumin, fructosamine, and 1,5-anhydroglucitol have been explored but found to be less attractive as they may not add to the existing value provided by the HbA1C [10]. However, as CKD progresses, HbA1C, glycated albumin, and fructosamine have been found to be decreasingly reliable. In CKD, glucose transporters decrease, leading to higher glycosuria. This results in impaired tubular reabsorption of 1,5-anhydroglucitol, leading to falsely low circulating 1,5-AG levels [11]. Table 1 describes the advantages and disadvantages of various tests used to diagnose and monitor T2D in those with CKD.

**Table 1:** Tests to diagnose and monitor diabetes. HD: hemodialysis; 1,5-AG: 1,5-anhydroglucitol; CGM: continuous glucose monitoring; OGTT: oral glucose tolerance test.

Test	Description	Advantages	Disadvantages
HbA1c	The most common test reflects average blood sugar control over 2–3 months. It predicts mortality in those with HD [12].	Easy to perform and standardized.	Accuracy declines with advanced CKD (G4-G5), particularly HD patients [13], and varies with drugs like protease inhibitors, [9] NRTIs, aspirin, vitamin C and E [14]. Falsely high A1c in metabolic acidosis false low A1c: high RBC turnover like Anemia, transfusion, Hemodialysis blood loss, ESA, and iron supplements [15,16]
Fructosamine	Less affected by factors that decrease HbA1c	It may be helpful in cases where HbA1c decreases.	Reliability decreases with advanced CKD. It may not add to the existing value provided by the HbA1C.
Glycated albumin	Measures sugar attached to albumin protein	May be useful in some CKD cases.	Unpredictable with inflammation, low albumin, and peritoneal dialysis. Frequent testing (every 2-4 weeks).
1,5-AG	Rapid glycemic changes and postprandial glucose were more accurate than HbA1c or fructosamine.	Not affected by red blood cell or protein turnover, unlike other markers [17-19].	Low levels seen in CKD can be misleading.
CGM	Measures interstitial fluid glucose levels, providing real-time data	May be useful for CKD patients at risk for hypoglycemia, offers personalized data and tailored plans [13].	Not yet approved for dialysis patients, costlier.
OGTT	Gold standard for diagnosing post-transplant diabetes [20]	Not recommended for routine monitoring.	Time-consuming test.

What is the Target HbA1C in CKD?

In CKD 5, where the estimated glomerular filtration rate (eGFR) is less than 15 ml/min/1.73 m^2^, it is crucial to have individualized target HbA1Cs ranging from 6.5% to ≤8% depending on patient factors like hypoglycemia (risk, awareness, availability of resources for self-management), cardiovascular disease, duration of diabetes, social support, and life expectancy [13]. Table 2 shows the target HbA1C for various stages of D, including when the patient is started on dialysis.

**Table 2:** HbA1C targets in specific populations with CKD. CKD: chronic kidney disease; eGFR: estimated glomerular filtration rate.

Stage of CKD		Target HbA1c	Rationale
CKD 4 (eGFR 15–30 ml/min)		6.5–8%	Similar to younger CKD 5 patients. [13]
CKD 5 (eGFR <15 ml/min)		Individualized: 6.5% to <8% [13]	-
Younger (<50 years) CKD 5 and dialysis		6.5–8%	Minimize long-term complications [13]
Older CKD 5 and dialysis with comorbidities		≤8%	Balance glycemic control with risk of hypoglycemia [13]
CKD 5 and dialysis	Comorbidity	6.5–8.0%	-
	Malnourishment	≥9.0% or <5.0%	Mortality is higher and worse survival [21]
	Compared to HbA1c of 6.5–7.4%	>8.5%	Mortality increases [22]
	Uremic patients initiating dialysis	≤5.4%	Mortality higher [22]

Management of DM in advanced CKD

Lifestyle Modifications

Lifestyle modifications include techniques to control DM and reduce co-morbidities like atherosclerotic cardiovascular disease (ASCVD) and dietary and physical activities that require a multidisciplinary team to succeed. Current recommendations regarding CVD risk factors in patients with DM and CKD include regular assessment every three to six months, which should include self-motivated lifestyle modifications. Self-motivation helps keep changes sustainable in managing DM at every stage of CKD.

Dietary recommendations emphasize diets high in vegetables, fruits, whole grains, plant-based proteins, limited processed meats, and refined carbohydrates. Protein should be tailored for the CKD stage and renal replacement therapy (RRT) modality. The suggested amount of daily protein for those with CKD who are not on dialysis and those on dialysis is 0.8 g/kg/day and 1.2 g/kg/day, respectively. Sodium intake should be restricted to <2 g/day [13,23]. Potassium intake is recommended at <4 g/day for CKD stage 3a and <2 g/day for CKD stage 5 [24]. Patients are also advised to stay on low-phosphorus-containing foods. Those with CKD may have diverse ethnic food patterns, and accredited nutrition providers who are involved should be culturally sensitive to socioeconomic factors when implementing dietary counseling.

Physical activity is recommended at a minimum of 150 minutes per week of moderate-intensity exercise tailored to individual cardiovascular and physical tolerance. Smoking cessation is essential, with physicians counseling patients to quit tobacco use and reduce exposure to secondhand smoke. Weight management is emphasized, particularly for obese patients with diabetes and CKD, with consideration given to eGFR levels [13].

Team-based integrated care is suggested to provide comprehensive care, focusing on risk evaluation and patient empowerment in managing kidney and heart risk factors in patients with diabetes and CKD [13].

Managing Other Comorbidities in DM and CKD

People with diabetes and CKD often have other health problems like morbid obesity, hypertension (HTN), proteinuria (albuminuria), hyperlipidemia, and increased ASCVD risk. Strategies to manage these conditions may include interventions that reduce weight, control blood pressure with specific medications, and lower lipids to recommended levels. 

In individuals with morbid obesity, bariatric weight reduction surgery offers potential benefits such as improvements in glycemic and lipid abnormalities and may slow the progression of CKD and improve kidney function [25,26]. However, there is limited prospective data on its effects on established CKD[i] or transplantation with regard to mortality, timing of surgery, and benefits [25,27-29].

Treating HTN with angiotensin-converting enzyme (ACE) inhibitors or angiotensin II receptor blockers (ARBs) is the first-line treatment. These medications should be gradually increased to the highest tolerated dose. It is important to avoid combining ACE inhibitors and ARBs or combining them with direct renin inhibitors, as this may cause hyperkalemia [13].

In those with hyperlipidemia, statin therapy is generally recommended for people with established heart disease (secondary prevention) and for preventing heart disease in certain groups, including adults over 40 with diabetes, those with CKD stages 1-4, and kidney transplant recipients (KTRs). However, statins may not appear to offer benefits for people on chronic dialysis who have competing causes for CVD besides hyperlipidemia [13].

Additional Medications for Heart and Kidney Protection

In addition to treating co-morbidities, specific medications have been shown to have specific benefits for those with DM and CKD, including glucagon-like peptide-1 receptor agonists (GLP-1 RAs), antiplatelet medications, and non-steroidal mineralocorticoid receptor antagonists (Ns-MRA).

GLP-1 RA can be used in type 2 diabetics with CKD who have not reached their blood sugar goals with metformin and sodium-glucose cotransporter 2 (SGLT2) inhibitors or who cannot take those medications as CKD advances. GLP-1 RAs may also be preferred for weight loss in obese patients with type 2 diabetes and CKD. They can increase the risk of low blood sugar when used with other diabetes medications like sulfonylureas (SU) or insulin, so dosage adjustments may be needed [13]. Antiplatelet therapies are recommended based on an individual’s risk of heart disease.

Ns-MRAs may be considered for type 2 diabetics with an eGFR above 25 ml/min/1.73 m^2^, who have normal potassium levels and persistent albuminuria despite maximum tolerated doses of RAS inhibitors. They can be combined with RAS inhibitors and SGLT2 inhibitors to treat diabetes and CKD. Steroidal MRAs are used for specific conditions but can cause side effects like high potassium or reduced kidney function [13].

Although medications that do not cause hypoglycemia are preferred and avoid frequent blood glucose monitoring, insulin is the most common medication used for DM in CKD 5. The available insulins are listed in Table 3 [30]. The transition from CKD 5 to dialysis is complex, with many medications and lifestyle adjustments. Patients who have DM and have been on insulin may find that as CKD progresses, they require less bolus insulin until dialysis is started. After the onset of RRT, removing uremic toxins may improve appetite and weight gain may be seen. Blood pressure and blood glucose levels fluctuate with the start of dialysis, and medication for both conditions may need to be adjusted frequently.

**Table 3:** Insulins use in advanced CKD. O: onset of action; P: peak of action; E: effective duration. Stage 3 = eGFR 30–60 ml/min, Stage 4 = eGFR 15–30 ml/min, Stage 5 = eGFR less than 15 ml/min. All % mentioned in the above table are % dose reduction of the suggested initial dose that may be used to start treatment, and titration may be needed [34-38].

Categories of insulin	Drugs name	Pharmacology	CKD3	CKD 4	CKD 5	HD	PD
Rapid-acting							
Rapid-acting insulin covers insulin needs for meals eaten at the same time as the injection. It may usually be used with longer-acting insulin.	Lispro (Humolog)	O: 15–30 min; P: 30–90 min; E: 3–5 hours	Yes	Yes	Yes	Yes	Yes
	Aspart (Novolog)	O: 10–20 min; P: 40–50 min; E: 3–5 hours	Yes	Yes	Yes	Yes	Yes
	Glulisine (Apidra)	O: 20–30 min; P: 30–90 min; E: 1–2 1/2 hours.	Yes	Yes	Yes	Yes	Yes
Short-acting							
Short-acting insulin covers insulin needs for meals eaten within 30–60 minutes.	Regular (R) or (Novolin)	O: 30 min; −1 hour; P: 2–5 hours; E: 5–8 hours	Yes	25%	50%	50%	50%
	Velosulin (Insulin pump- based on Insulin used)	O: 30 min; −1 hours; P: 1–2 hours; E: 2–3 hours	Yes	25%	50%	50%	50%
Intermediate-acting							
Intermediate-acting insulin covers insulin needs for about half the day or overnight. This type of insulin is often combined with a rapid- or short-acting type.	NPH (N)	O: 1–2 hours; P: 4–12 hours; E: 18–24 hours	Yes	25%	50%	50%	50%
Long-acting							
No peak effect delivered at a steady level. Long-acting Insulin covers insulin needs for about one full day. This type is often combined, when needed, with rapid- or short-acting insulin.	Insulin Glargine (Basaglar, Lantus, Toujeo)	O: 1–1 1/2 hours; P: No peak time; E: 20-24 hours	Yes	25%	50%	50%	50%
	Insulin Detemir (Levemir)	O: 1–2 hours; P: 6–8 hours; E: Up to 24 hours	Yes	25%	50%	50%	50%
	Insulin Degludec (Tresiba)	O: 30–90 min; P: No peak time; E: 42 hours	Yes	25%	50%	50%	50%
Premixed*							
These products are generally taken two or three times a day before mealtime. *Premixed insulins combine specific amounts of intermediate-acting and short-acting Insulin in one bottle or an insulin pen. (The numbers following the brand name indicate the percentage of each type of insulin.)	Humulin 70/30	O: 30 min; P: 2–4 hours; E: 14–24 hours	Yes	25%	50%	50%	50%
	Novolin 70/30	O: 30 min; P: 2–12 hours; E: Up to 24 hours.	Yes	25%	50%	50%	50%
	Novolog 70/30	O: 10–20 min; P: 1–4 hours; E: Up to 24 hours	Yes	25%	50%	50%	50%
	Humulin 50/50	O: 30 min; P: 2–5 hours; E: 18–24 hours	Yes	25%	50%	50%	50%
	Humalog mix 75/25	O: 15 min; P: 30 min to 2 1/2 hours; E: 16–20 hours	Yes	25%	50%	50%	50%

Insulin for Glycemic Control

Individualized dosing is necessary for the use of short-, intermediate-, and long-acting insulin in CKD. Total insulin requirements decrease by 25% when GFR falls below 50 ml/min/1.73 m^2^ and decrease by 50% when GFR falls below 10 ml/min/1.73 m^2^ [31-33]. Upon initiation of dialysis, peripheral insulin resistance may improve, further reducing insulin requirements [34,35].

All insulin doses are to be individualized for patients with CKD 4 and 5. Rapid-acting insulin after the meal should be given for patients with CKD 4-5, hemodialysis (HD), and PD, and poor appetite will help match the postprandial peak [36,37]. Short-acting insulin is considered to be close to physiologic insulin and can be used in advanced CKD. It needs to be combined with continuous glucose monitoring (CGM) or self-monitoring of blood glucose (SMBG) [36]. There is no clear directive for the use of long-acting insulins for patients on dialysis, either HD or PD. Long-acting insulin may require a dose reduction to avoid hypoglycemia [38]. Premixed insulins offer the convenience of twice-daily dosing, have limited flexibility, may require injection at fixed times, need consistent food intake, and may provide better glucose control [36]. In PD with overnight cycler use, better control is achieved with premixed insulin, especially if dialysate concentrations are changed [37]. There continues to be no clear directive on long-acting insulin for glycemic control for those on dialysis. While some patients and physicians are comfortable with long-acting insulin in a steady state, situations that change caloric intake, like hospitalization, may add challenging variables, and the patient may best be treated with shorter-acting insulin in that setting (Table 3).

Insulin in Hemodialysis

The dialysis process can result in glycemic changes. Historically, glucose was added to hemodialysis solutions to prevent acute hypoglycemia during the treatment as glucose diffuses out of the patient’s blood into the dialysate. Modern hemodialysis solutions contain less glucose (100 mg/dL) compared to the past (200 mg/dL) to reduce vagal tone and better counteract intradialytic hypotension [39]. The glucose concentration of current dialysis solutions allows the movement of glucose from or to the blood based on the gradient between plasma and dialysis solution. In those on HD, both hyperglycemia and insulin levels can be reduced via HD on the treatment days [40]. This may require reducing insulin doses by 25% on dialysis days to avoid hypoglycemia [41].

Insulin in Peritoneal Dialysis

In peritoneal dialysis (PD), the modality and solution may impact blood glucose differently; automated nocturnal, compared to continuous ambulatory PD, has less glucose exposure. The commonly used solution is carbohydrate-based with varying doses to allow for greater osmotic removal of volume (1.5%, 2.5%, and 4.25% have 15, 25, and 42.5 g of dextrose per liter). In addition to the PD prescription and the patient’s peritoneal membrane characteristics, different amounts of glucose may be absorbed. While some may add insulin to the PD solution, it is infrequently used due to the varying absorption of insulin from the solution, the significant loss of insulin due to adherence to the bag (up to 30% of the insulin), and the risk of contamination [42]. A new finding of uncontrolled hyperglycemia should raise concern for peritonitis, as inflammation increases glucose absorption from the PD solution. Non-glucose-based solutions used in PD include icodextrin, which is associated with glucose avoidance while maintaining fluid removal and may improve survival [43].

Insulin in Kidney Transplant

In the immediate kidney transplantation (KT) period, insulin is the most commonly used anti-glycemic agent. In the perioperative period for kidney transplantation, medications like steroids, calcineurin inhibitors, and stress may increase insulin resistance and the need for insulin. Continuous glucose monitoring may allow the recognition of atypical episodes of hyperglycemia, which would be missed otherwise, and recurrent or de-novo DM nephropathy can occur sooner in the transplanted kidney [44,45].

Other Glycemic Therapies

A common suggestion by nephrologists is to dose medication for eGFR or dialysis, as medications may not be appropriately cleared and could result in the buildup of metabolites, leading to complications. Most medications for T2DM can be used for early CKD, with a few needing dose adjustments. As CKD progresses, fewer categories of medications for T2DM are available for use. In addition to the accumulation of metabolites, the efficacy for glycemic control of medications like SGLT2i may vary based on kidney function.

Medications that were avoided with kidney transplantation are now being cautiously used, including SGLT2i, GLP-1RA, and dipeptidyl peptidase IV inhibitors (DPP4i), with care to avoid dehydration, which may result in a usual dip in renal function, which may be confused with rejection. In addition, patients need to be monitored for urinary tract infections [46]. The cardiovascular benefits of these medications are less clear in the transplant population.

In general, there are several groups of non-insulin anti-glycemic medications that are commonly used in the treatment of T2D. Biguanides decrease hepatic gluconeogenesis. Gastrointestinal side effects are noted after the patient has used the medication for four years and may need vitamin B12 and folate supplementation. Lactic acidosis is a serious complication in patients with an eGFR <45 ml/min [47]. Metformin can be used in stable patients with preserved renal function as a first-line agent in kidney transplant recipients with an eGFR of more than 30 ml/min/1.73 m^2^ and a BMI greater than 25 kg/m^2^ [48,49]. Patients should be instructed to refrain from taking their medication if they are ill, infected, or dehydrated [50].

SGLT2 inhibitors can improve CVD and CKD outcomes in those without and with early CKD [51,52]. While some may suggest continuing use until the initiation of RRT, additional medications may be needed for glycemic control [53,54]. In dialysis, it has been theorized that there may be receptors for SGLT2i in the heart and vasculature, which may benefit cardiovascular morbidity and mortality, which is currently being studied. SGLT2i are less effective for glycemic control if eGFR <45 ml/min; additional medication for glycemic control is needed. They are suggested to be used until eGFR >20 ml/min [13]. SGLT2i has limited data in KT patients, but it is available and shows a similar effect in non-transplanted patients with T2DM or post-transplant diabetes mellitus (PTDM). There is a lack of studies on KTR without diabetes [55].

GLP1 receptor agonists are injectables with stronger hypoglycemic and extra-pancreatic effects. Benefits for those with DM and eGFR > 14 ml/min include a reduction in the progression of atherosclerotic vascular disease and albuminuria [56]. GLP1RA is preferred for renal protection over DPP-4i. GI side effects may confound the symptoms of peritonitis. In transplant, GLP-1 RA is recommended by most guidelines as a second-line alternative to SGLT2i after metformin in managing T2DM, especially with CVD, CV risk factors, or CKD. Human GLP1RAs like liraglutide, semaglutide, and dulaglutide are not excreted via kidneys and can be used up to eGFR 15 ml/min, and data are insufficient below that [36]. They can be used with caution in HD, and semaglutide and dulaglutide are used as once-weekly injections [57]. Only liraglutide was studied in PD. Interaction with transplant medication mycophenolate alters GI absorption of the drug. There are limited data on GLP1RA in KT, but small studies show they are safe and can be carefully used [57]. Non-human GLP1RA, like exenatide and lixisenatide, are eliminated by the kidney and contraindicated below eGFR <30 ml/min [58].

Tirzepatide is a GIP receptor and GLP-1 receptor agonist, leading to significantly improved glycemic control in type 2 diabetics and significant weight reduction. There is insufficient data on HD and PD, but it may be safe to use as hepatic or renal dysfunction does not affect metabolism. There are no studies of its effect on diabetic patients with KT [59].

Dipeptidyl peptidase 4 (DPP-4) inhibitors, also called gliptins, work by inhibiting the breakdown of incretins like glucagon-like peptide-1 (GLP-1) and gastric inhibitory peptide (GIP). Incretins are short-lived hormones that are secreted after a meal and stimulate insulin release. DPP-4 inhibitors extend the effect of incretins without causing hypoglycemia or weight gain [35].

Sitagliptin may have some benefit in protecting the peritoneal membrane. Alogliptin appears to be safe, with some benefits, in small studies in KT [60]. Linagliptin does not interact with calcineurin or mTOR inhibitors and is considered safe to use in KT [61]. Others, like anagliptin, omarigliptin, trelagliptin, and teneligliptin, are not available in the US. They likely have some benefit from class action, but no studies have looked at these specific categories [35,60,61].

Sulfonylureas are insulin secretagogues that are generally metabolized by the liver and may cause hypoglycemia. SU metabolites, like glyburide, may accumulate in CKD, and first-generation SU is contraindicated. Some second-generation SUs can be used with caution in CKD. Glipizide is preferred in RRT due to its shorter action [62,63].

Meglitinides are fast-acting insulin secretagogues taken before food. They decrease glucose levels after a meal, which can result in hypoglycemia. To avoid hypoglycemia, they should be started at the lowest dose with slow titration.

Alpha-glucosidase inhibitors work by slowing digestion and the absorption of carbohydrates after a meal. These medications are avoided with an eGFR <30 ml/min. Data for and against use in HD, with KDOQI recommending against use [64,65]. Miglitol is mostly cleared by the kidney and is avoided in CKD [37].

Thiazolidinediones work by decreasing hepatic glucose production and do not result in hypoglycemia; this medication can result in fluid retention, may increase the risk of heart failure, and may be used with caution [37]. It may be safe for use in PD. Table 4 shows how the above medications are used in those with T2D and advanced CKD.

**Table 4:** Non-insulin therapy for advanced CKD. *Not enough evidence. ^Reduced dose and use with caution.

Categories	Medication	CKD 3	CKD 4	CKD 5	HD	PD	KT
Biguanides [50]	Medication	CKD 3	CKD 4	CKD 5	HD	PD	KT
	Metformin	Yes^^ ^[50]	No [37]	No	No	No	Yes, if eGFR >30 ml/min [13]
	Buformin* [66]	No	No*	No*	No*	No*	Maybe* if eGFR >60 ml/min
SGLT2i	Medication	CKD 3	CKD 4	CKD 5	HD	PD [67]	KT [55,60]
	Dapagliflozin	Yes	Yes	No	No	No	Yes
	Empagliflozin	Yes	Yes	No	No	No	Yes [68]
	Canagliflozin	Yes	No	No	No	Yes* [69]	Yes*
	Others: Ipragliflozin Luseogliflozin Tofogliflozin	Yes	No*	No*	No*	No*	No*
GLP 1 RA (tides)	Medication	CKD 3	CKD 4	CKD 5	HD [57]	PD	KT [70]
	Human GLP-1 derived [71]						
	Liraglutide	Yes	Yes	Yes*	Yes	Yes [72]	With caution*
	Dulaglutide	Yes	Yes	Yes*	Yes	Yes*	With caution*
	Semaglutide (injectable and oral)	Yes	Yes	Yes*	Yes	Yes	With caution*
	Non-human GLP-1 derived [58]						
	Exenatide	Yes	No	No	No	No	No*
	Lixisenatide	Yes	No	No	No	No*	No*
Dual GLP-1/GIP receptor antagonist	Medications	CKD 3	CKD 4	CKD 5	HD	PD	KT
	Tirzepatide [59]	Yes	Yes	Yes	With caution*	With caution*	With caution*
DPP4-I (gliptins) [35]	Medications	CKD 3	CKD 4	CKD 5	HD	PD [35]	KT [60]
	Sitagliptin	Yes^	Yes^	Yes^	Yes^	Yes*	Yes^
	Saxagliptin	Yes^	No	No	Yes^	Yes^	Yes^
	Alogliptin	Yes^	Yes^	Yes^	Yes^	Yes^	With caution*
	Vildagliptin	Yes^	No	No	With caution*	No*	With caution*
	Linagliptin	Yes^	Yes^	Yes^	Yes	Yes^	Yes [61]
	Others: Anagliptin Omarigliptin Trelagliptin teneligliptin	Yes^*	No*	No*	Yes^	No*	No*
Sulfonylurea (SU)	Medications	CKD 3	CKD 4	CKD 5	HD [62]	PD [35]	KT
	Glipizide	Yes	Yes^ [37]	Yes^ [37]	Yes*^	Yes [63]	Yes [73]
	Glimepiride	Yes^ <60 eGFR [37]	Yes ^	No	No	No	No
	Gliclazide	Yes^ <40 eGFR	No	No	No	No	No
	Glyburide	Yes^	No	No	No	No	No
	Gliquidone tolbutamide acetohexamide tolazamide glyclopyramide glipalamide chlorpropamide	No	No	No	No	No	No
Meglitinides (glinides) fast acting insulin secretagogues	Medications	CKD 3	CKD 4	CKD 5	HD	PD	KT
	Nateglinide	Yes^ <60 eGFR	No	No	Yes* but dialyzed out [37]	No	Yes [74]
	Repaglinide	Yes^ <30 eGFR	Yes	Yes*	Yes* [63]	No	Yes
	Mitiglinide	Yes	Yes	Yes	Yes [61]	No*	Yes^
Alpha-glucosidase inhibitors	Medications	CKD 3	CKD 4	CKD 5	HD	PD	KT
	Acarbose	No	No	No	No*	No	No
	Miglitol	No	No	No	No*	No	No
Thiazolidinediones	Medications	CKD 3	CKD 4	CKD 5	HD	PD	KT
	Pioglitazone [37]	Yes	Yes	Yes	Yes	Yes	No*

Special situations

In those with advanced CKD, special situations may be encountered that may change T2D management, as in the hospitalized patient or the patient with hyperosmolars who can quickly have glucose removed with dialysis. Table 5 shows specific conditions in those with T2D and advanced CKD and the change in treatment management that is specific for that condition.

**Table 5:** Special situations in treatments for diabetes and CKD.

Situation	Subset	Management change
Hyperkalemia treatment using insulin	Hypoglycemia	In advanced CKD, 13–18% of cases are due to factors like limited blood sugar and impaired insulin metabolism in kidney problems [75,76].
Hyperkalemia treatment using insulin	Monitoring potassium and glucose	The potassium-lowering effect lasts 2 hours, but blood sugar monitoring is needed for up to 3 hours after treatment to avoid prolonged hypoglycemia [77].
Diabetic gastroparesis	-	This may result in a mismatch between diabetic treatment and meals and blood glucose fluctuations that are difficult to control, especially with changes with RRT.
Burnt out DM	-	CGM range may be useful to clarify further treatment
Medications leading to fluid gains/fluid retention of advanced CKD	-	Nonsteroidal anti-inflammatory drugs, calcium channel blockers, corticosteroids, thiazolidinediones, sodium containing medication. May need adjustment in diuretics or ultrafiltration if on dialysis.
Hospitalized patients	-	With T2DM and CKD, eGFR 15-45 ml/min/1.73 m^2^ - using once-daily glargine and three-times daily glulisine at half the dose (0.25 units/kg vs 0.5 units/kg) showed similar glycemic control with significantly less hypoglycemia in the group with the lower weight-based dose [38].
	Continuous renal replacement therapy	Glucose can be continuously removed, and that can result in euglycemic diabetic ketoacidosis [78]
Hyperosmolar diabetes with dialysis	-	Treat with insulin and limit fluids as there is limited urine output and hence nominal volume depletion. Dialysis may be able to correct the electrolyte imbalances, remove glucose, and establish an euvolemic state, and careful dosing of Insulin may prevent hypoglycemia.
Preoperative	GI procedures like colonoscopy	Insulin may need to be reduced due to reduced blood glucose.
Perioperative	Surgery	Nothing by mouth orders before surgery, especially abdomen surgery: will need medication titration.
Infection	Sepsis	Blood glucose is lowered due to consumption. May need a lower dose of Insulin.
Infection	Peritonitis	More glucose may be absorbed than may be consumed due to infection. The use of rapidly acting lower-dose Insulin with more liberal targets may be needed for greater metabolic stability.
Infection	Prevention of infection	Intensive glucose control with Insulin improved in those with multiorgan failure, hospital mortality, bloodstream infection, and acute kidney injury needing dialysis [79].
Pancreatic islet cell transplantation	-	T1DM recommended to have pancreatic transplantation with kidney transplantation
Perioperative kidney transplant	Diagnosing T2DM	Perioperative period after KT has significant metabolic instability due to drugs like steroids and blood transfusion affecting HbA1c, and the diagnosis is made using oral glucose tolerance testing [80].
Perioperative kidney transplant	New-onset diabetes after transplant/post-transplant diabetes	In the setting of glucocorticoid use, or a subset of patients who had hepatitis C and were treated with tacrolimus.
Perioperative kidney transplant	SGLT2i	Usually avoided early after transplantation with the highest levels of immunosuppression, which may exacerbate the chances of UTI while on SGLT2i and volume depletion status.
Perioperative kidney transplant	CGM	Option in situations where HbA1C does not correlate to daily glycemic monitoring results or where there is use of medication that could lead to hypoglycemia [13].

## Conclusions

Treating T2DM in those with advanced CKD is complex and should include a multi-disciplinary team approach while being patient-centric. In patients who have been on chronic dialysis or transplantation, new T2D medications can be cautiously tried with benefit. For those who are in a clinical transition, the use of insulin and prior medication with careful dosing may be needed. Flexibility and frequent monitoring of medication use to match caloric intake help with the prevention of hypoglycemia.
